# Non-Woven Haemostatic Agent Mimicking Perigraft Abscess Following Thoracic Aortic Surgery

**DOI:** 10.3390/reports9010040

**Published:** 2026-01-28

**Authors:** Ali Ansaripour, Arian Arjomandi Rad, Andrea D’Alessio, Antonios Kourliouros

**Affiliations:** Department of Cardiothoracic Surgery, Oxford Heart Centre, John Radcliffe Hospital, Oxford, OX3 9DU, UK

**Keywords:** oxidised cellulose haemostat, aortic surgery, abscess

## Abstract

**Background and Clinical Significance**: Absorbable haemostatic agents such as Surgicel^®^ Fibrillar are useful adjuncts to control post-surgical bleeding in cardiac surgery. The material is purposefully left in situ and it slowly degrades over time. Previous publications, mainly in general and gynaecological surgery, have demonstrated that these materials can mimic gangrenous infection, abscesses, anastomotic leak, and early tumour recurrence in imaging studies. These findings can often lead to unnecessary re-interventions or re-operations. The number of reports in the cardiothoracic surgical field is limited. **Case Presentation**: We report a 45-year-old man who underwent aortic valve replacement and ascending aorta and hemiarch replacement. In this case, Surgicel^®^ Fibrillar was used to optimise graft contouring, contributing to postoperative imaging appearances that initially raised concern for infection. The patient was conservatively managed given his stable clinical picture and focused review of CT images with the knowledge of location of Surgicel^®^ Fibrillar. Repeat CT scan after 2 weeks showed a significant reduction in collection size and complete resolution of air bubbles within the collection. **Conclusions**: It is important for cardiothoracic surgeons and radiologists to be aware of the early CT appearances of haemostatic agents to minimise erroneous diagnosis of postoperative complications leading to unnecessary interventions. This case highlights a diagnostic pitfall in postoperative imaging, where retained absorbable haemostatic material may mimic serious infective complications and lead to unnecessary re-intervention if operative and radiological findings are not carefully correlated.

## 1. Introduction and Clinical Significance

Oxidised regenerated cellulose (ORC), (Surgicel^®^, Johnson and Johnson Medical, Arlington, TX, USA), has been increasingly utilised in cardiac surgery to control bleeding and occasionally to adjust the shape and position of aorto-coronary bypass grafts. Surgicel^®^ comes in sterile-knitted fabric or fibrillar forms and can be sutured or cut to fit the appropriate surgical field. Published literature across multiple surgical specialties has reported that Surgicel^®^ remnants can mimic an abscess cavity or early tumour recurrence [1]. In cardiothoracic surgery, there have been very few case reports involving the misdiagnosis of Surgicel^®^ remnants as abscess cavity, or thrombus [2,3]. In some of these cases patients underwent emergency re-operations which only revealed sterile retained Surgicel^®^. This case focuses on erroneous radiological suspicion of perigraft abscess following ascending aorta and hemiarch replacement, and the successful avoidance of unnecessary re-intervention.

Clinical Significance: This case illustrates the diagnostic pitfall caused by retained ORC in thoracic aortic surgery, emphasising the need for radiologic–surgical correlation to prevent unnecessary invasive procedures.

## 2. Case Presentation

A 45-year-old man was referred to our cardiac surgical service due to mixed aortic valve disease with bicuspid appearance and 55 mm dilated ascending aorta from the sinotubular junction extending to the level of the proximal arch. His left ventricular function and dimensions were preserved, and his main complaint was exertional breathlessness (NYHA II–III) and one episode of chest pain. The patient was a non-smoker with no history of diabetes, immunosuppression, or other significant comorbidities. He underwent mechanical aortic valve replacement, and ascending aorta and hemiarch replacement. The procedure was carried out with right axillary and right femoral arterial and two-stage right atrial cannulation, and systemic cooling to 24 °C nasopharyngeal temperature. The aorta was cross clamped during cooling and the heart arrested initially with retrograde cardioplegia followed by selective coronary ostial cardioplegia.

Following routine aortic valve replacement with 27 mm ATS (ATS Medical, Inc., Minneapolis, MN, USA) mechanical valve and completion of the proximal anastomosis at the level of sinotubular junction with 30 mm Gelweave graft (reinforced with Teflon strip), circulatory arrest was initiated with selective antegrade cerebral perfusion through the right axillary and left carotid arteries. A bevelled open distal anastomosis with Teflon strip was performed to extend into the mid arch inferiorly while preserving the origin of the brachiocephalic artery. After reperfusion and separation from cardiopulmonary bypass, we used Surgicel^®^ Fibrillar to enhance the concavity of the aortic graft due to increased native aortic annular angulation.

**Figure 1:**
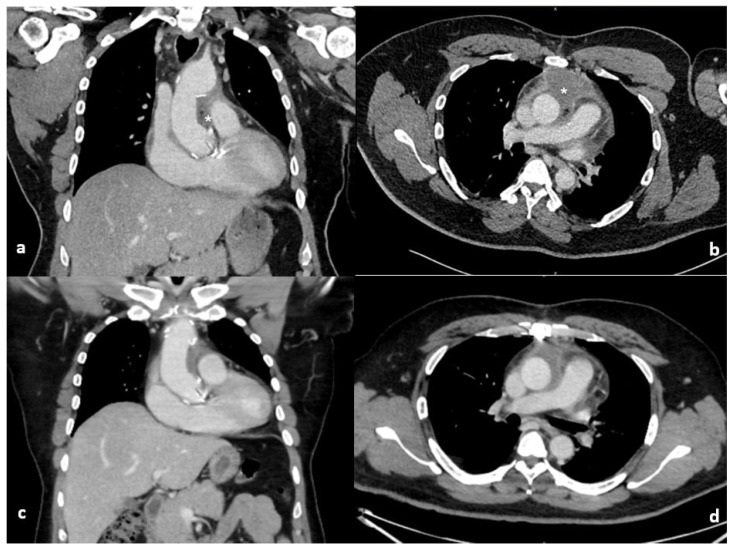
Coronal and axial computed tomography of the chest on the 10th (**a**,**b**) and 27th (**c**,**d**) post operative days. The initial large loculated collection with blebs of air and enhancing walls (*) surrounding the ascending aorta has improved with conservative management on repeat imaging.

Postoperatively, inflammatory markers rose in keeping with a postoperative inflammatory response. C-reactive protein peaked on postoperative day 3 and subsequently demonstrated a sustained downward trend. White cell count peaked later, on postoperative day 8, but remained discordant with the patient’s clinical status, as he was afebrile, haemodynamically stable, and without features of sepsis. Serial blood cultures were negative. Table 1 demonstrates the timeline of inflammatory markers, imaging and management. This temporal dissociation between biochemical markers and clinical findings, together with radiological features and knowledge of intraoperative Surgicel^®^ placement, supported a conservative management strategy. Empirical intravenous amoxicillin–clavulanate was commenced in response to rising inflammatory markers and later transitioned to oral therapy. All blood cultures obtained during this period were negative. An enhanced CT, Figure 1, revealed a 6.5 × 5.5 × 10 cm loculated collection with blebs of air and enhancing walls surrounding the ascending aorta, mainly within the aortopulmonary space. Given the stable clinical picture, the surgical correlation with the use of Surgicel^®^ Fibrillar in this location, a decision was made to conservatively manage the patient on oral antibiotics for an additional week in the community. A repeat CT scan was arranged on the 27th postoperative day, which revealed the reduction in size of periaortic fluid collection and complete resolution of locules of gas. The inflammatory markers at this point were normalised with WCC and CRP of 9.03 × 10^9^/L and 25.9 mg/L, respectively. Subsequent surveillance CT imaging at 6 months and 18 months postoperatively confirmed stable aortic graft appearances with no evidence of residual collection or infection.

## 3. Discussion

Oxidised regenerated cellulose products are used to control bleeding by providing a matrix for platelet adhesion and aggregation. They are applied dry to the surgical site and most frequently undergo complete dissolution within 2–4 weeks [4]. The CT appearances of Surgicel^®^ and its resemblance to a postoperative abscess have been described in the literature [2]. Some radiographic features may help differentiate the haemostatic material from an abscess. Surgicel^®^ could appear as a unifocal collection of gas with linearly arranged gas bubbles and no air–fluid levels, while a postoperative abscess typically shows air–fluid levels and scattered air bubbles [2]. These appearances are thought to result from trapped air within the material and the acidic by-products of cellulose degradation rather than active infection.

In the present case, Surgicel^®^ Fibrillar was not used primarily for haemostasis but to enhance the concavity and orientation of the ascending aortic graft, a less commonly reported indication. This non-standard application may involve a greater volume or altered configuration of material, potentially prolonging its radiological persistence. Awareness of this practice and close radiological–surgical correlation were central to avoiding unnecessary re-intervention.

When an early CT scan is required within the time that it takes for Surgicel^®^ Fibrillar to become absorbed (minimum of 7–14 days), it is advisable to inform the reporting radiologist about the presence and location of these materials. A prospective study of imaging in patients following pelvic surgery demonstrated that knowledge by the reporting radiologist of the intraoperative use of Surgicel^®^ Fibrillar can decrease the rate of misdiagnosing postoperative abscess, thus avoiding unnecessary interventions [5]. Incorporating specific annotations by the surgical team regarding the use and precise location of haemostatic agents like Surgicel^®^ during the procedure can significantly aid radiologists in their image analysis. By establishing these practices, healthcare professionals can enhance diagnostic accuracy, thereby reducing the risk of unnecessary interventions and optimising patient care outcomes in the postoperative period.

### 3.1. Contextualising the Literature with This Case

Our case underscores the diagnostic challenge posed by retained ORC, particularly Surgicel^®^ Fibrillar, in postoperative imaging. The material’s radiologic resemblance to abscesses or other complications necessitates a nuanced approach to diagnosis that integrates clinical, radiologic, and intraoperative insights. A detailed review of recent literature highlights key themes that inform the interpretation and management of such findings, which we explore in the context of cardiothoracic surgery.

### 3.2. Radiologic Mimicry and Diagnostic Challenges

Retained ORC often presents on imaging with characteristics that mimic pathological findings, such as abscesses, hematomas, or tumour recurrence. Liu (2016) documented delayed absorption of Surgicel^®^ in post-thyroidectomy patients, where the material mimicked pseudoabscesses on imaging up to 47 months postoperatively. This study highlighted the diagnostic challenge posed by ORC in patients undergoing routine imaging, particularly when the material is retained for longer than expected [6].

Similarly, Zhang (2015) described a case where intraovarian Surgicel^®^ mimicked acute pathology in a young woman, prompting unnecessary diagnostic concerns. Importantly, the imaging abnormalities resolved spontaneously with conservative management. This case underscores the critical importance of clinical correlation to avoid unnecessary intervention, a theme mirrored in our case [7].

In our case, the CT findings initially suggested a perigraft abscess, which could have justified surgical exploration. However, the absence of systemic infection signs, such as fever or hemodynamic instability, and the gradual improvement in inflammatory markers (e.g., C-reactive protein and white cell count) led to a decision to manage the patient conservatively. This approach aligns with best practices described in the literature, where misdiagnosed ORC-related findings often lead to unwarranted reoperations, as reported by Sharifi (2015) in renal surgery and Alameer (2023) in thyroid surgery [8,9].

### 3.3. The Role of Radiological–Surgical Collaboration

Effective communication between radiologic and surgical teams is paramount in preventing misdiagnosis of ORC-related findings. Jenkins (2020) highlighted how retained Surgicel^®^ could appear as an abscess-like echogenic collection on ultrasound following laparoscopic surgery. The authors emphasised the value of detailed surgical documentation and preoperative communication with radiologists to ensure accurate interpretation of postoperative imaging [10].

Our case exemplifies the importance of this collaboration. During the postoperative period, the surgical team recognised that Surgicel^®^ Fibrillar had been placed near the ascending aortic graft, prompting careful reinterpretation of imaging findings. This awareness allowed the team to consider alternative explanations for the apparent abscess, leading to a decision of conservative management. As Franceschini (2021) noted, including specific annotations about ORC placement in operative notes significantly improves radiologic interpretations and reduces the risk of unnecessary interventions [11].

### 3.4. Bioabsorption Timeline and Clinical Implications

A critical consideration in interpreting postoperative imaging involving ORC is the bioabsorption timeline. Mathew (2018) observed that Surgicel^®^ could mimic pelvic abscesses on CT scans shortly after surgery, but the imaging findings resolved spontaneously over weeks as the material was absorbed [12]. In our case, a repeat CT scan two weeks after the initial imaging demonstrated significant reduction in the size of the fluid collection and complete resolution of air bubbles, confirming that the radiologic findings were related to retained Surgicel^®^ and not an actual abscess.

Interestingly, Franceschini (2019) cautioned that improper placement or excessive retention of ORC can occasionally lead to adverse outcomes, such as granulomatous reactions or incomplete absorption, further complicating imaging and clinical management [13]. This highlights the importance of using only the minimum amount of ORC necessary to achieve haemostasis, as well as closely monitoring postoperative imaging when the material is left in situ.

### 3.5. Implications for Cardiac Surgery

Implications for cardiac surgery are increasingly recognised, although ORC-related complications have been predominantly studied in general and gynaecological surgery. The scarcity of documented cases in cardiac surgery emphasises the need for enhanced awareness among cardiothoracic surgeons and radiologists regarding ORC’s imaging characteristics. To address this gap, our case provides an example specific to thoracic aortic surgery, complementing the following case reports:

Regragui et al. presented a case of a 56-year-old woman with suspected early-onset prosthetic valve endocarditis following aortic valve replacement. Imaging revealed a periaortic echogenic mass misinterpreted as an abscess. In this case the presence of acute fever in the early postoperative period prompted concern for early prosthetic valve endocarditis. Although blood cultures were sterile, the initial clinical presentation justified escalation until an alternative source of infection was identified and surgical intervention confirmed retained Surgicel [14].

Kaneyuki (2018) reported a 65-year-old woman undergoing a redo-Bentall operation, where hypodense mediastinal masses and air bubbles on CT were mistaken for mediastinitis. This case described a high-risk redo operation which lowers the threshold for intervention. Emergency reoperation revealed no infection but retained Surgicel^®^, highlighting the challenges posed by Surgicel^®^ remnants and the necessity for accurate documentation to avoid unnecessary surgeries [15].

Martínez (2021) highlighted a different diagnostic challenge, demonstrating persistent false-positive FDG uptake on PET/CT up to 22 months after aortic valve replacement due to Surgicel. In that case, positive blood cultures and systemic symptoms initially supported the diagnosis of possible endocarditis, but careful integration of echocardiographic findings and operative history avoided unnecessary surgical intervention [16].

The present case differs from these reports in several important respects. Our patient remained afebrile and haemodynamically stable, blood cultures were persistently negative, and inflammatory markers demonstrated an early plateau followed by sustained down-trending. In addition, the CT appearance—characterised by a loculated perigraft collection with linear gas locules and no air—fluid level—was consistent with retained oxidised regenerated cellulose. Crucially, explicit knowledge of the intraoperative use and anatomical location of Surgicel^®^ Fibrillar enabled accurate radiological–surgical correlation.

These factors collectively supported a conservative management strategy with close surveillance, resulting in radiological resolution and durable long-term clinical stability. This comparison underscores that unnecessary re-intervention may be avoided when clinical stability, biochemical trends, imaging features, and operative details are considered together rather than in isolation.

### 3.6. Practical Approach to Suspected Perigraft Collections in the Presence of ORC

The present case highlights the importance of a structured, multidisciplinary approach when postoperative imaging raises concern for perigraft infection in patients in whom oxidised regenerated cellulose has been used. Conservative management may be considered in selected cases when the patient is clinically stable, blood cultures are negative, inflammatory markers demonstrate early stabilisation or downward trends, and imaging features are compatible with retained ORC rather than abscess formation.

**Figure 2:**
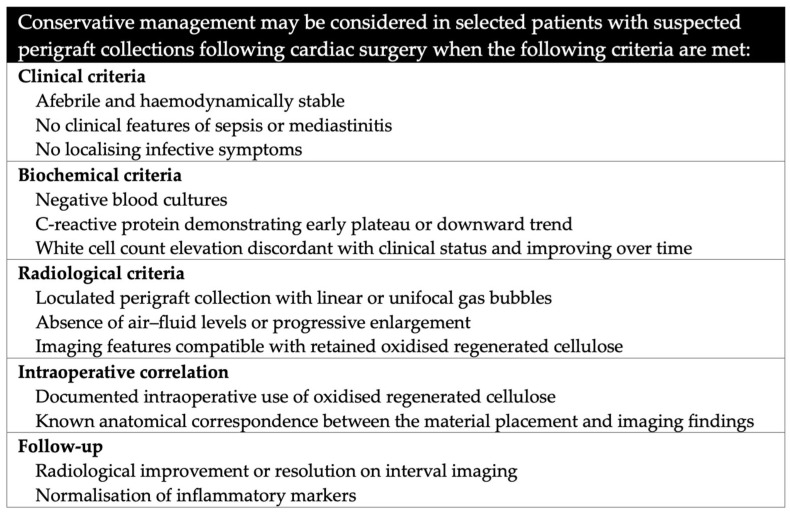
Clinical and radiological criteria supporting conservative management when oxidised regenerated cellulose is used.

Clinical and radiological criteria supporting conservative management are summarised in Figure 2. This pragmatic framework may assist cardiothoracic surgeons, intensivists, and radiologists in avoiding unnecessary re-intervention while maintaining patient safety.

These cases collectively underline the diagnostic challenges associated with ORC use in cardiac surgery and the need for multidisciplinary collaboration to prevent misdiagnoses.

## 4. Conclusions

Our case demonstrates the diagnostic complexities associated with retained ORC, particularly in the unique context of cardiac surgery. The literature reviewed provides critical insights into the radiologic mimicry of ORC, the importance of interprofessional collaboration, and the role of bioabsorption timelines in postoperative imaging. Together, these lessons emphasise the need for meticulous documentation, radiologic awareness, and clinical correlation to optimise patient outcomes and minimise unnecessary interventions.

## Figures and Tables

**Table 1:** Postoperative timeline of inflammatory markers, imaging, and management. POD; post operative day, f/u; follow up.

	WCC (×10^9^/L)	CRP (mg/L)	Key Events/Clinical Status
Pre-op	7.82	5	Baseline preoperative bloods
POD 1	10.5	60	Postoperative inflammatory response
POD 2	11.56	294	Rising inflammatory markers
POD 3	14.96	360 (peak)	No fever, haemodynamically stable
POD 4	12.16	306	CRP downtrend begins
POD 5	12.6	233	Continued clinical stability
POD 6	19.5	240	Empirical IV amoxicillin–clavulanate commenced
POD 7	20.7	263	Afebrile, blood cultures negative
POD 8	22.65 (peak)	262	WCC peak with stable CRP
POD 9	16.9	176	Biochemical stabilisation
POD 10	17.3	154	No clinical signs of sepsis
POD 11	16.0	120	CT chest: loculated perigraft collection with linear gas
POD 13	14.2	69	Downward inflammatory trend
POD 14	14.25	57	Discharged on oral antibiotics
POD 27	9.03	25.9	Repeat CT: marked reduction, gas resolved
6 months f/u	–	–	Surveillance CT: no collection
18 months f/u	–	–	Surveillance CT: stable graft, no infection

## Data Availability

The corresponding author will provide the anonymised data that supports the conclusions of this study upon a reasonable request, following ethical and data protection rules.

## Abbreviations

The following abbreviations are used in this manuscript:

ORC	Oxidised Regenerated Cellulose
CT	Computed Tomography
WCC	White Cell Count
CRP	C-reactive Protein
NYHA	New York Heart Association
PET	Positron Emission Tomography
